# Enhanced Phospholipase A2 Group 3 Expression by Oxidative Stress Decreases the Insulin-Degrading Enzyme

**DOI:** 10.1371/journal.pone.0143518

**Published:** 2015-12-04

**Authors:** Daishi Yui, Yoichiro Nishida, Tomoko Nishina, Kaoru Mogushi, Mio Tajiri, Satoru Ishibashi, Itsuki Ajioka, Kinya Ishikawa, Hidehiro Mizusawa, Shigeo Murayama, Takanori Yokota

**Affiliations:** 1 Department of Neurology and Neurological Science, Graduate school of Medicine, Tokyo Medical and Dental University, Tokyo, Japan; 2 Department of Bioinformatics, Medical Research Institute, Tokyo Medical and Dental University, Tokyo, Japan; 3 Center for Brain Integration Research, Tokyo Medical and Dental University, Tokyo, Japan; 4 National Center Hospital, National Center of Neurology and Psychiatry, Tokyo, Japan; 5 Department of Neuropathology, Tokyo Metropolitan Institute of Gerontology, Tokyo, Japan; Ehime University Graduate School of Medicine, JAPAN

## Abstract

Oxidative stress has a ubiquitous role in neurodegenerative diseases and oxidative damage in specific regions of the brain is associated with selective neurodegeneration. We previously reported that Alzheimer disease (AD) model mice showed decreased insulin-degrading enzyme (IDE) levels in the cerebrum and accelerated phenotypic features of AD when crossbred with alpha-tocopherol transfer protein knockout (*Ttpa* ^-/-^) mice. To further investigate the role of chronic oxidative stress in AD pathophysiology, we performed DNA microarray analysis using young and aged wild-type mice and aged *Ttpa* ^-/-^ mice. Among the genes whose expression changed dramatically was Phospholipase A2 group 3 (Pla2g3); Pla2g3 was identified because of its expression profile of cerebral specific up-regulation by chronic oxidative stress *in silico* and in aged *Ttpa* ^-/-^ mice. Immunohistochemical studies also demonstrated that human astrocytic Pla2g3 expression was significantly increased in human AD brains compared with control brains. Moreover, transfection of HEK293 cells with human Pla2g3 decreased endogenous IDE expression in a dose-dependent manner. Our findings show a key role of Pla2g3 on the reduction of IDE, and suggest that cerebrum specific increase of Pla2g3 is involved in the initiation and/or progression of AD.

## Introduction

Alzheimer disease (AD) is the most common age-related neurodegenerative disease characterized by the deposition of amyloid-beta (Aβ), accumulation of hyperphosphorylated Tau containing neurofibrillary tangles, reactive astrocytes and loss of synapses and neurons [1]. While there are several kinds of rare familial early-onset AD caused by gene mutations, the majority are sporadic cases with various etiologies. However, accumulation of Aβ is the initial event of AD according to the Aβ hypothesis [2,3]. The strongest risk factor for AD is aging so its prevalence increases exponentially with age [4,5]. Aβ accumulation is seen even in the preclinical AD brains; however, it is more robust in mild cognitive impairment (MCI) brains and is most severe in AD brains [6]. One of AD model mouse strains that overexpresses the Swedish mutant form of human Aβ precursor protein (*APPsw*) also shows age-related Aβ accumulation in the brain [7,8].

Another early pathological event in AD is increased oxidative stress [9–13], which contributes to membrane damage, deterioration of synaptic plasticity and neuronal cell death [14], suggesting that oxidative stress promotes the initiation and/or progression of the disease. We previously generated α–tocopherol transfer protein (α-TTP) knockout (*Ttpa* ^-/-^) mice [15]. *Ttpa* ^-/-^ mice show marked lipid peroxidation in the brain because of a lack of α–tocopherol and are highly valuable as a model of chronic oxidative stress to the brain. By crossing AD transgenic model mice with *Ttpa* ^-/-^ mice, we obtained double mutant *APPsw*/*Ttpa* ^-/-^ mice, which showed increased Aβ deposits in the cerebrum and enhanced cognitive dysfunctions to be compared with *APPsw* mice. The accumulation of Aβ and cognitive deficits in the double mutant mice were ameliorated with α–tocopherol supplementation [16,17]. Together with the fact that lipid peroxidation may be a major cause for aging of the brain [18], oxidative stress and aging are major contributors to AD pathogenesis.

Although many studies of oxidative stress on neurons and glial cells have been done using a cell culture system, the precise molecular and cellular mechanisms underlying AD pathology *in vivo* have not been fully elucidated. The goal of this study is to clarify the molecular mechanisms of how aging and/or oxidative stress accelerates AD pathophysiology. The region specific pathophysiological changes, Aβ deposits and neurofibrillary tangles in cerebral cortex but not in cerebellum, were well characterized using the postmortem AD brains [1]. *APPsw* mice also showed the distribution of Aβ mainly in cerebrum but not in cerebellum [19]. This lesion specific increase of Aβ accumulation in AD brains let us suppose a hypothesis that some molecules, which alter its expression only in the cerebrum but not in the cerebellum, have important role on AD pathophysiology. We performed DNA microarray analysis using *Ttpa* ^-/-^ mice and found Pla2g3 expression was significantly induced only in the cerebral cortex of *Ttpa* ^-/-^ mice but not in the cerebellum. Chronic oxidative stress is thought to be highly relevant to the slow progression of AD pathophysiology, thus, specificity of Pla2g3 induction in chronic oxidative condition was confirmed using *Ttpa* ^-/-^ mice as chronic oxidative stress model and acute oxidative stress model mice induced by traumatic brain injury and stroke. In this study, we thus provide evidence that Pla2g3 plays a role in brain region-specific changes in AD pathology under chronic oxidative stress.

## Ethical statement

The Institutional Review Board (IRB) of Tokyo Medical and Dental University approved this study. We obtained written informed consent for research use of human paraffin sections from relatives at the time of autopsy. The Animal Experiment Committee of Tokyo Medical and Dental University approved animal experiments. The Ethics Committee of Tokyo Metropolitan Institute of Gerontology approved human study.

## Materials and Methods

### Animals

Generation of *Ttpa* ^-/-^ mice was previously described [15]. Wild-type and *Ttpa* ^-/-^ mice were fed on a normal (36 mg of α-tocopherol/kg), α-tocopherol-supplemented (600 mg of α-tocopherol/kg), or α-tocopherol-deficient diet until the time of use. For the ischemic mouse model, male C57Bl/6 (18 weeks old) mice were used for the surgical procedure for left permanent middle cerebral artery occlusion [20] and used for the histological examinations on day 7 after surgery. For the traumatic brain injury model, postnatal day 2 (P2) mice were subjected to cryogenic injury as described previously [21]. Briefly, P2 pups were anesthetized by spontaneous inhalation of 2% isoflurane, and the parietal skull was exposed through a scalp incision. A metal probe (1.5-mm diameter) cooled by liquid nitrogen was placed on the right skull (0.5-mm anterior and 1.2-mm lateral to the bregma) for 30 seconds. Following this procedure, the scalp was immediately sutured and the pups were returned to the home cage and the pups were fixed after 3 days. The Animal Experiment Committee of Tokyo Medical and Dental University approved animal experiments.

### RNA isolation

RNA was isolated using mirVana mRNA isolation kit (Ambion) according to the manufacturer’s protocol. RNA concentration was quantified with NanoDrop Spectrometer (NanoDrop technologies) at a wavelength of 260nm and an Agilent 2100 Bioanalizer was used to evaluate its integrity.

### Microarray

Gene expression in cerebral cortex and cerebellum of mice were determined using Agilent chips. To ensure higher quality results in gene expression data, we conducted microarrays on 4 mice per group. Young mice were 2 months old and the other aged mice were 29 months old at the time of use. Data were standardized using global normalization and processed by R-program. An absolute fold change threshold of greater than 1.5 was required to be considered for further analyses. Expression values were in log2 scale. The original data analysed in this publication have been deposited in NCBI’s Gene Expression Omnibus and are accessible through GEO series accession number GSE75047.

### Cell culture

Culture conditions of HEK293 and TR-AST cell lines were described previously [22,23]. Primary cultured astrocytes were prepared from postnatal day 0 Wistar rats. Culture preparation was performed as described previously [24]. Primary astrocytes were cultured for 2 weeks before the oxidative stress examination. HEK293 cells were used for transient transfection of human Pla2g3 expression vector (Origene). For the oxidative stress examinations, TR-AST cells were treated with 2 mM hydrogen peroxide for 6 hours, and primary astrocytes were treated with 0.4 mM hydrogen peroxide for 6 hours, otherwise, indicated in the figure legend. The culture plates of the cells were placed on ice at the harvest and rinsed with ice-cold PBS then followed by RNA extraction procedure.

### qRT-PCR

Reverse transcription of RNA to cDNA was performed with the Roche Reverse transcription kit using the following conditions: 25°C for 10 min, 37°C for 50 min, and 85°C for 5 min. Quantitative PCR was performed subsequently on the resulting DNA using Roche SYBR green master mix and the primers. All the primers were designed using Roche universal design tool and pairs of primers met with highest criteria score were selected. The primer sequences are provided in S1 Table. The PCR thermal cycling conditions are follows: denaturing step at 95°C for 5 min, followed by 45 cycles of 95, 60, 72°C for 10, 10 and 10 sec, respectively. Specificity of each primer set was checked by the melting curve analysis upon quantitative PCR. The delta-Ct for mouse Pla2g3 and mouse GAPDH were subjected to simple t-test to yield the estimation of delta-delta-Ct. The relative expression changes were calculated with amplification efficiency approximating 2.

### Western blotting

The temporal lobe was dissected and homogenized in lysis buffer containing 1% Triton X-100, 0.1% SDS, 20 mM Tris pH 7.5, 150 mM NaCl, 1 mM EDTA and protease inhibitors cocktail (Roche). Tissue was then sonicated using Poly Tron 2100 (Kinematica AG), and insoluble material was removed from the protein extracts by centrifugation at 10,000rpm for 20 min at 4°C. Protein content in the supernatant was quantified with the BCA assay (Thermo). Equivalent amounts of total protein were separated in 4–20% gradient SDS-PAGE gels (Wako) and transferred to nitrocellulose membranes (Millipore). Membranes were blocked with Tris-buffer saline containing 0.1% Tween 20 and 5% nonfat milk for 1 hour, then incubated with primary antibody to Pla2g3 (1:1000; Origene), alpha-tubulin (1:2000; MBL), and were then visualized with appropriate HRP-conjugated secondary antibodies.

### Histology

Mice were intracardially perfused with ice-cold PBS followed by 4% (w/v) ice-cold paraformaldehyde (PFA) in PBS. The dissected brains were then post-fixed overnight with 4% PFA at 4°C. Three mice were used in each group until otherwise stated in the figure legend. Human paraffin sections of superior frontal gyrus (Brodmann area 9) were provided from Tokyo Metropolitan Geriatric Hospital and Institute of Gerontology under the material transfer agreement. We obtained written informed consent for research use from relatives at the time of autopsy. The Ethics Committee of Tokyo Metropolitan Institute of Gerontology approved human study.

### Immunohistochemistry and antibodies

For DAB staining, the fixed brains were paraffin embedded, coronally sectioned, and analyzed using Olympus BX53 microscope. For immunofluorescent staining, vibratome sections were analyzed by confocal microscopy (LSM-510 META; Zeiss) [25]. DAB and immunofluorescent staining were performed as previously described [26]. Antibodies used for immunostaining were as follows: anti-Pla2g3 (1:250; Origene) and anti-GFAP (1:500; Sigma).

### Quantification of Pla2g3-immuno reactive cells

To quantify Pla2g3-immunoreactive (IR) cells, six images of superior frontal gyrus including the molecular and external granular layers were captured per paraffin section. Images were taken at × 200 magnification and analyzed with ImageJ software to quantify Pla2g3-IR cells. The result of quantification was expressed as number of cells/450 × 325 μm.

### Statistical analysis

All data values are presented as the mean ± S.E.M. At least four mice per genotype were used per experiments. Student’s t tests were applied to data with two groups of samples. One-way ANOVAs were used for comparisons of data with more than two groups and were followed by the Turkey-Kramer *post hoc* test for significance. A *p* value of <0.05 was considered statistically significant. Data were analyzed using GraphPad Prism for Windows.

The Institutional Review Board (IRB) of Tokyo Medical and Dental University approved this study.

## Results

### DNA microarray analysis in mice under oxidative stress

The causes of Aβ accumulation in sporadic AD are not fully understood, but oxidative stress is involved in this process. To investigate the role of chronic oxidative stress on AD pathophysiology *in vivo*, we employed *Ttpa* ^-/-^ mouse line that has marked oxidative stress due to vitamin E deficiency in the brain. Our previous reports showed the accelerated accumulation of Aβ in the cerebrum of *APPsw*/*Ttpa* ^-/-^ double mutant mice [16], and the double mutant mice showed earlier and more severe cognitive dysfunction than *APPsw* mice [17]. Consequently, *Ttpa* ^-/-^ mouse is thought to be suitable to study the role of oxidative stress in AD pathophysiology.

**Fig 1:**
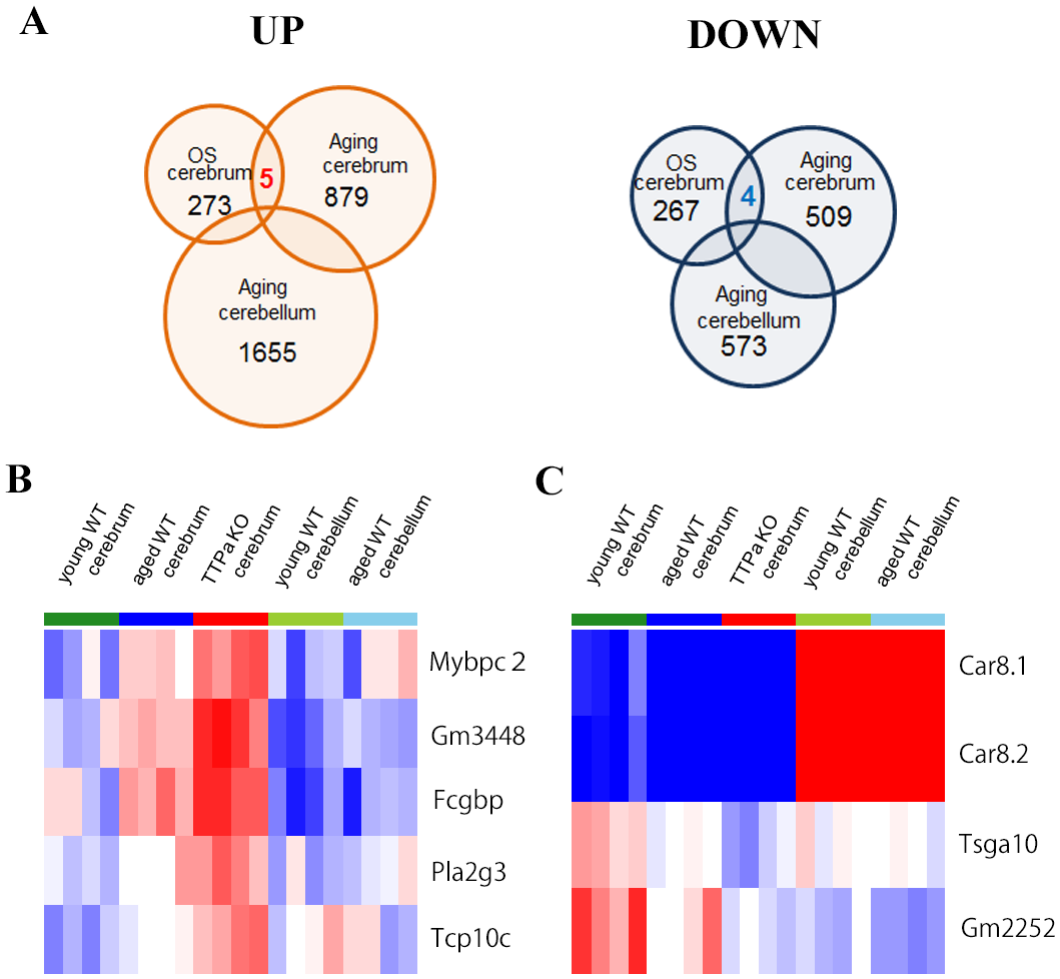
Microarray analysis of cerebrum specific genes regulated by oxidative stress and aging. *A*, Each circle is indicating the numbers of either up or down regulated genes by oxidative stress, aging in the cerebrum and in the cerebellum. Five genes were up-regulated and four genes were down regulated by both oxidative stress and aging in the cerebrum without significant changes in the cerebellum. *B*, The heat maps of five genes up-regulated and *C*, four genes down-regulated by oxidative stress and aging. Red indicates up-regulated genes and blue indicates down-regulated genes, while white indicates no significant change in expression. The color gradients indicate the intensity of expression. The data shows the results of four mice per each group. Abbreviation used; OS; oxidative stress.

To find the candidate genes implicated in the acceleration of AD pathophysiology, we performed microarray analysis using *Ttpa* ^-/-^ mouse cerebrum and cerebellum (Fig 1A). First, we compared 29-month-old *Ttpa* ^-/-^ mouse cerebrum with age-matched wild-type mouse cerebrum and found 273 up-regulated genes and 267 down-regulated genes by oxidative stress. Next, we compered 29-month-old wild-type mouse cerebrum with 2-month-old wild-type mouse cerebrum and found 879 up-regulated genes and 509 down-regulated genes by aging in the cerebrum. Among those genes, 13 genes were up-regulated both by oxidative stress and by aging in the cerebrum and 6 genes were down-regulated in the same manner. Further, we compered 29-month-old wild-type mouse cerebellum with 2-month-old wild-type mouse cerebellum and found 1655 up-regulated genes and 573 down-regulated genes by aging in the cerebellum. We excluded 454 genes that were up-regulated by aging both in the cerebrum and in the cerebellum and 51 genes that were down-regulated in the same manner from this study. Then, we finally found nine genes which were differently expressed by oxidative stress and aging only in the cerebrum but not in the cerebellum. Five genes, Fcgbp, Gm3448, Mybpc2, Pla2g3 and Tcp10c, are significantly up-regulated in the cerebrum both by normal aging and by oxidative stress without any significant changes in the cerebellum by normal aging (Fig 1B). Also, four genes, Car8.1, Car8.2, Gm2252 and Tsga10, are significantly down-regulated in the cerebrum both by normal aging and by oxidative stress without any significant changes in the cerebellum by normal aging (Fig 1C). Among those genes with significantly changed expression by array analysis, Pla2g3 is the only one that has been reported to be involved in Alzheimer’s disease and we first focused on this gene.

### Induction of Pla2g3 expression in cerebrum by oxidative stress

**Fig 2:**
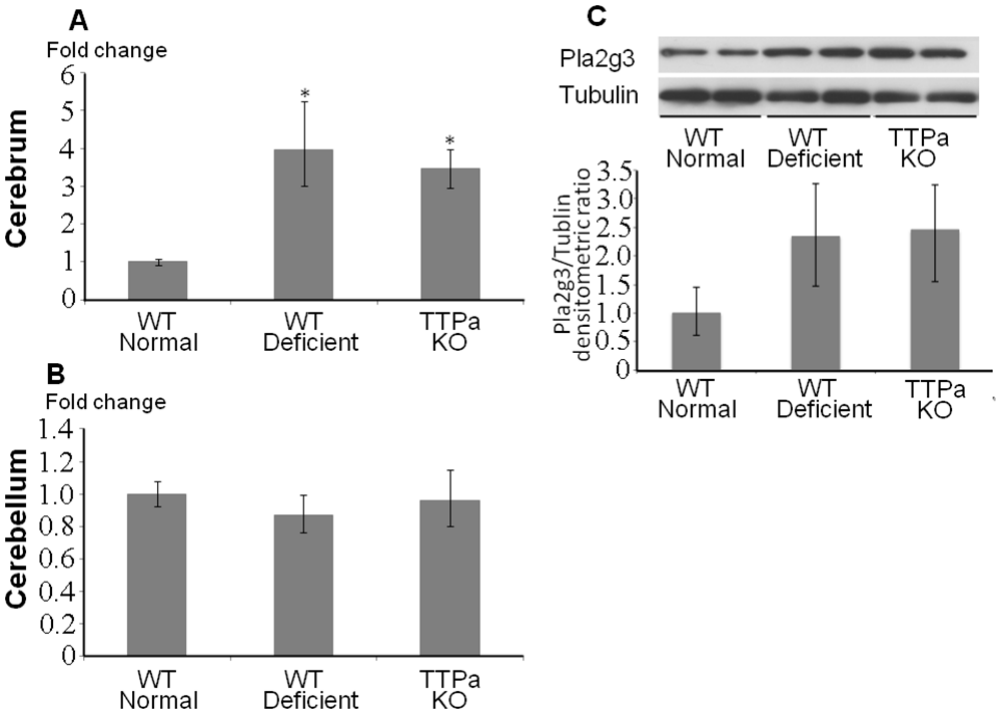
Induction of Pla2g3 expression in cerebrum by oxidative stress. *A*, *B*, Quantitative RT-PCR results of Pla2g3 in cerebrum and cerebellum are shown. Fold changes to the aged wild-type mice on normal diet are indicated. n = 4 in each group. *C*, Western blots for Pla2g3 and alpha-tubulin are shown. Abbreviation used; WT normal; wild type 29 months old mice fed on normal diet, WT deficient; 29 months old wild type mice fed on vitamin E deficient diet, ttpKO deficient; 29 months old *Ttpa* ^*-/-*^ mice fed on vitamin E deficient diet. *p<0.05.

We confirmed that Pla2g3 mRNA was significantly increased in the cerebrum of wild-type and *Ttpa* ^-/-^ mice fed with vitamin E deficient diet compared to wild-type mice fed with normal diet by qRT-PCR (Fig 2A). In contrast, the expression of Pla2g3 mRNA was not affected under oxidative stress in cerebellum (Fig 2B). The increased Pla2g3 protein expression in cerebral cortex by oxidative stress was also confirmed by western blot (Fig 2C). Thus, Pla2g3 could be one of candidate genes involved in this region specific acceleration of Aβ accumulation by oxidative stress and we studied its role further in the following experiments.

### The expression of Pla2g3 in astrocytes is dramatically increased by oxidative stress

**Fig 3:**
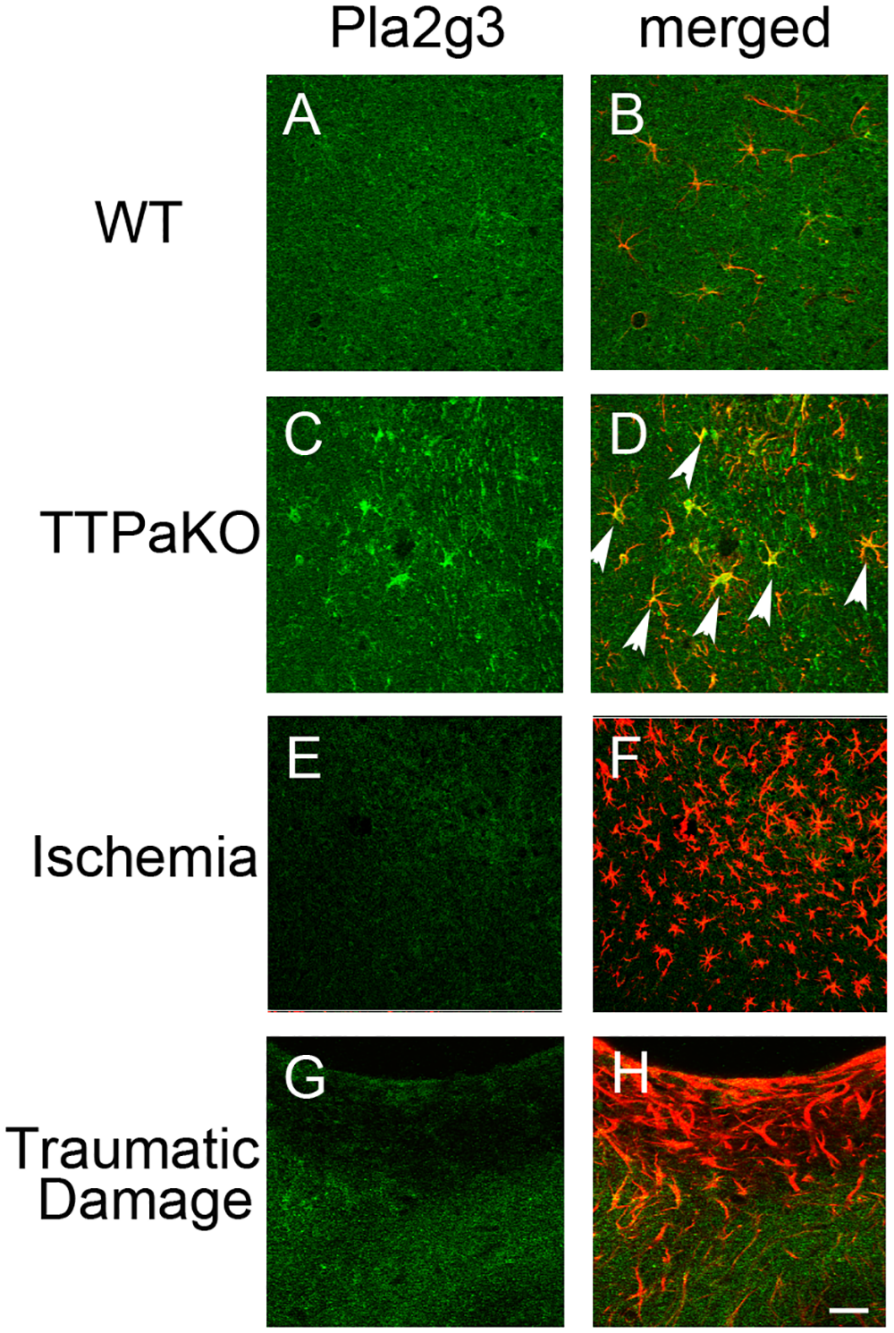
Astrocytic expression of Pla2g3 by chronic oxidative stress. Double immunostaining of Pla2g3 (green) and GFAP (red) in 29 months old wild type mouse (A, B), 29 months old *Ttpa* ^-/-^ mouse (C, D), Ischemic site (E, F) and traumatic injury site (G, H) of cerebral cortex of wild type mice. Arrow heads indicate the strong Pla2g3 expression in astrocytes of *Ttpa* ^-/-^ cortex. Scale bar: 50 μm.

It has been reported that Pla2g3 is expressed in variety of cell types in many organs including brain [27]. To characterize the expression pattern of Pla2g3 in the brain by oxidative stress, we carried out immunofluorescence study. Pla2g3 signals were weak and disperse and were mostly co-localized with astrocyte marker, GFAP, in the cerebral cortex of wild-type mice (Fig 3A and 3B). On the other hand, the active astrocytes were observed in the equivalent area of *Ttpa* ^-/-^ mice cerebral cortex and the intense Pla2g3 signals were induced in those active astrocytes (Fig 3C and 3D arrowheads). Pla2g3 induction was not observed in an acute oxidative stress accompany with the gliosis by ischemia (Fig 3E and 3F) or traumatic brain injury (Fig 3G and 3H) to the cerebral cortex, suggesting that the chronic oxidative stress is a key condition to induce Pla2g3 expression in astrocytes *in vivo*.

### Overexpression of Pla2g3 reduces IDE expression

**Fig 4:**
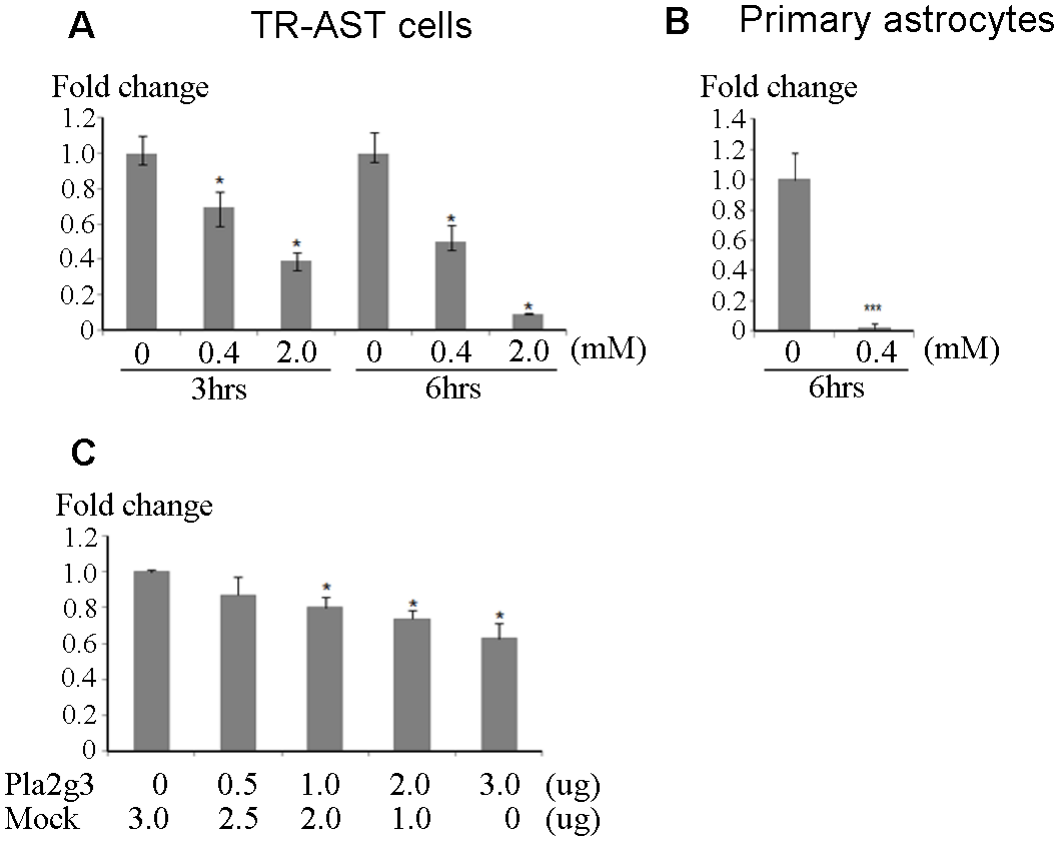
Overexpression of Pla2g3 reduces IDE expression. *A*, *B*, Quantitative RT-PCR result of IDE in TR-AST cells and rat primary astrocytes. TR-AST cells and primary astrocytes were treated with hydrogen peroxide in indicated conditions. Fold changes to the non-treated cells as controls are indicated. *C*, Quantitative PCR results of IDE in Pla2g3 transfected HEK293 cells. Human Pla2g3 was transiently expressed and cells were harvested after 48 hours of transfection. Fold changes to the mock control are indicated. *p<0.05, ***p<0.001.

We previously reported that the clearance of Aβ from brain was decreased in *Ttpa* ^-/-^ mice compared with wild-type mice, mainly due to the decreased expression level of IDE [17]. Surprisingly, IDE expression was significantly decreased in TR-AST cells after exposure to hydrogen peroxide in both a time-dependent and dose-dependent manner (Fig 4A) and IDE expression of rat primary astrocytes also showed extremely high sensitivity to hydrogen peroxide (Fig 4B).

To further study the possible role of Pla2g3 in the accumulation of Aβ, we overexpressed Pla2g3 using a cell culture system to examine whether Pla2g3 affects the endogenous expression level of IDE. Because of low transfection efficiency of primary astrocytes and TR-AST cells, we employed HEK293 cells to yield high transfection efficiency that also express IDE endogenously. In HEK293 cells, IDE expression was significantly reduced by the expression of Pla2g3 dose-dependently after 48 hours (Fig 4C). This indicates that Pla2g3 might be implicated in the accumulation of Aβ through the suppression of IDE expression.

### Increased expression of Pla2g3 in AD patients

**Fig 5:**
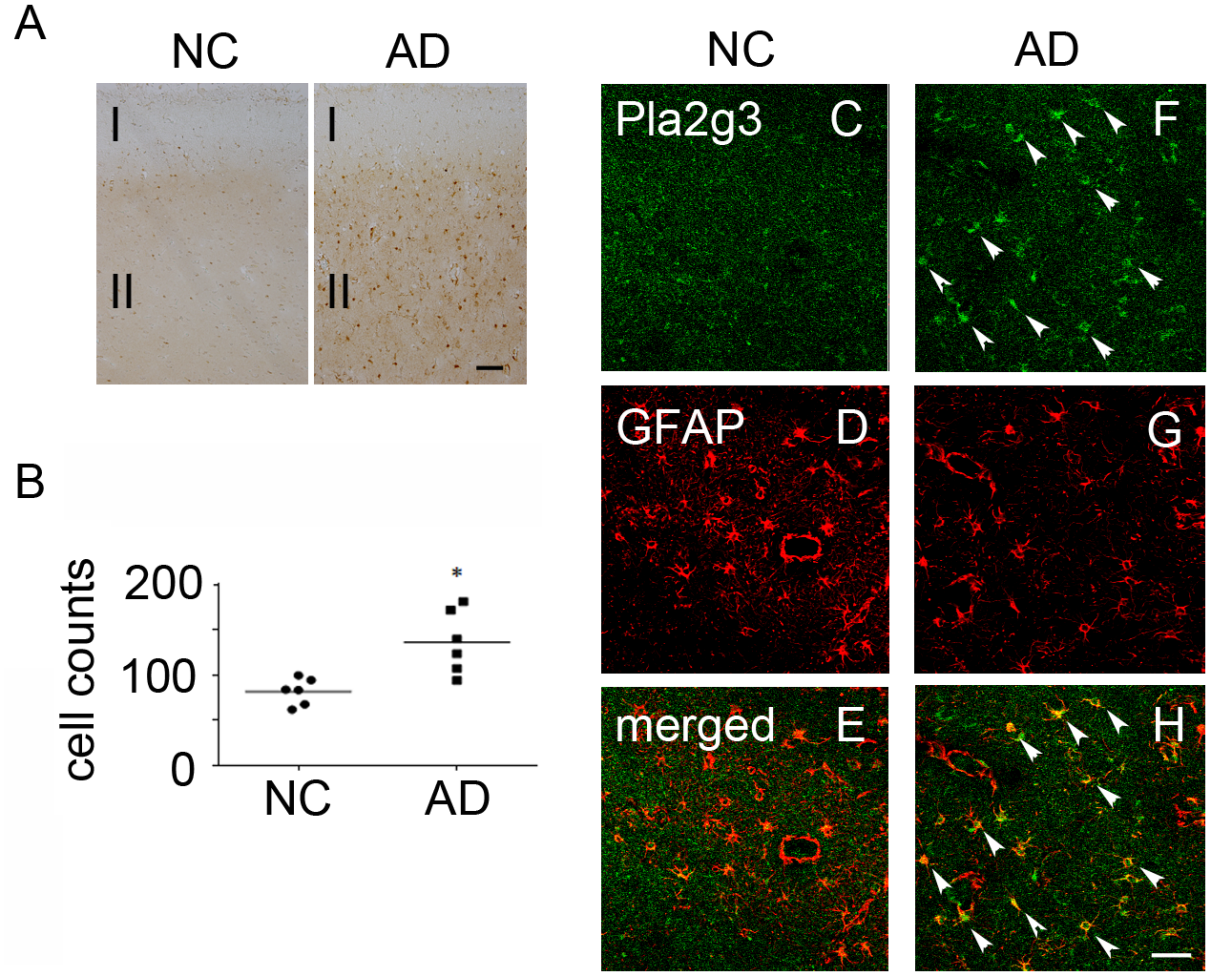
Increased expression of Pla2g3 in frontal cortex of AD patients. *A*, Pla2g3 expression in normal control brain and AD brain. The images are representative of 6 cases in each group. Frontal cortex was stained with anti-Pla2g3 antibody. *B*, Analysis of Pla2g3-positive cell numbers in frontal cortex. *p<0.05 *C-H*, Double immunostaining of Pla2g3 and GFAP in normal control brain (C-E) and AD brain (F-H). Arrowheads indicate the intensive staining of Pla2g3 in astrocytes of AD brain. Scale bars: *A*, 300 μm; *C*, 50 μm. Abbreviation used; NC; normal control, AD; Alzheimer’s disease patient, I; Molecular layer, II; External granular layer.

We next examined the expression of Pla2g3 in AD patients. DAB staining using anti-Pla2g3 antibody showed only weak and diffused staining in frontal cortex of normal control brains (Fig 5A). On the other hand, in AD brains, the intense Pla2g3-immunoreactivity was observed in the external granular layer (Layer II) and the signals were spread to the deeper layers. The quantitative analysis of Pla2g3-immunoreactive cells demonstrated significant increase in cerebral cortex of AD (Fig 5B). Immunofluorescence studies revealed only disperse staining of Pla2g3 in normal controls (Fig 5C) as compared to the intensive Pla2g3 staining co-localized with GFAP signals observed in AD patients (Fig 5F and 5H arrow heads), indicating that Pla2g3 expression is promoted mostly in astrocytes of AD patients. Together with the earlier findings, Pla2g3 might be involved in the initiation and/or progression of AD through IDE suppression in astrocytes.

## Discussion

The lipids of the brain, such as phosphatidylcholine, are integrated in the phospholipid membrane of brain cells and at one time were believed to have only a structural role. PLA2s are a diverse family of enzymes that catalyze the cleavage of fatty acids at the sn-2 position of glycerophospholipids to generate lysophospholipids and free fatty acids including arachidonic acids, which are important second messengers in signal transduction [28,29]. PLA2 are categorized into three groups, Ca^2+^-dependent cytosolic PLA2 (cPla2), Ca^2+^-independent PLA2 (iPla2), and Ca^2+^-dependent secretory PLA2 (sPla2) which includes Pla2g3. It has been established that brain lipids participate both in the function and in structure of brain cells and PLA2s play a key role not only in physiological events but also in pathological events such as neurogenesis [30–32] and neuronal cell death [33–38].

In the present study, we demonstrated that Pla2g3 expression is increased in cerebral cortex but not in cerebellum by chronic oxidative stress with vitamin E deficiency. Pla2g3 is expressed both in neurons and astrocytes but oxidative stress-induced Pla2g3 expression is predominantly in astrocytes in the cerebrum. It is particularly noteworthy that this increased Pla2g3 expression is absent in the astrogliosis induced by the ischemia or traumatic brain injury to the cerebral cortex, indicating that not the acute oxidative stresses but the chronic oxidative stress is the essential factor for induction of Pla2g3 expression *in vivo*. Moreover, Pla2g3 expression is not associated with the formation of reactive astrocytes. This brain region-specific astrocytic induction of Pla2g3 may be due to the regional differences of vulnerability to the accumulation of oxidative stress during aging. In fact, cerebellum has been reported to be more resistant to oxidative stress than hippocampus and frontal cortex of human brain [39], which provides a rational explanation for the cerebral cortex specific induction of Pla2g3 we observed. Additionally, Pla2g5 and iPla2 did not increase significantly and Pla2g2e was not detectable by oxidative stress *in vivo* despite a marked simultaneous increase in TR-AST cells after hydrogen peroxide treatment (S1 Fig), suggesting that the regulation of PLA2s gene expression varies among the family to support the complex lipids metabolism in the brain and each PLA2 might execute distinct cellular processes.

PLA2s participate in a variety of physiological processes, including remodeling of cellular membranes, signal transductions and host defense. Pla2g3 is expressed in a wide variety of organs but is most abundant in testis and brain [28]. Human Pla2g3 has been suggested to be involved in atherosclerosis in apo-E deficient mice [40], and its overexpression causes spontaneous skin inflammation in human Pla2g3-transgenic mice [41]. Recent study has shown that Pla2g3 plays a key role in maturation of mast cells *in vivo* [42], indicating Pla2g3 modulates gene expression profile in certain cells. However, the function of Pla2g3 in the brain has not been fully understood. Bee venom Pla2g3 has been demonstrated to cause apoptosis in rat primary cortical neurons [43] although human Pla2g3 has pro-survival effects on PC12 cells and promotes its neuron-like differentiation [33]. In the current study, human Pla2g3 transfected HEK293 cells also did not show any apoptotic signs after 48 hours of transfection (data not shown). Moreover, despite the profound induction of apoptosis observed in the region of ischemia and traumatic brain injury site of the mice, no induction of Pla2g3 expression was observed in this time course. Together with previous reports, our results indicate that Pla2g3 is not directly involved in apoptosis so that there may be a functional difference between bee venom and human Pla2g3. It is of note we found that human Pla2g3 is capable of reducing IDE expression *in vitro*. Expression of many genes including proteases, cytokines and chemokines are affected by Pla2g3 and those changes are considered to be mediated by bioactive lipid mediators [44], thus, IDE expression could be influenced by the lipid mediator pathways acting directly downstream of Pla2g3. Although other PLA2s are possibly capable of reducing IDE expression because of the same enzymatic activities they possess, they are less likely to be involved in AD pathophysiology since either their expression was undetectable by qRT-PCR or unchanged by chronic oxidative stress (S1 Fig).

AD is a progressive neurodegenerative disorder, which leads to the most common causes of dementia. The alteration of amyloid precursor protein (APP) processing, the aggregation and/or clearance of Aβ can be considered crucial in the progression of AD. We previously reported that the production of Aβ was not changed but the clearance of Aβ from brain was prominently decreased in *APPsw*/*Ttpa* ^-/-^ double mutant mice causing accelerated Aβ accumulation compared with *APPsw* mice, indicating that oxidative stress has a profound effect on Aβ clearance. One of enzymes involved in Aβ homeostasis is IDE, which degrades Aβ and plays a role on Aβ efflux from brain [45]. In *Ttpa* ^-/-^ mice, IDE expression is markedly decreased compared to wild type mice. We demonstrated that overexpression of human Pla2g3 suppresses IDE expression in a dose-dependent manner. Therefore, we presume that Pla2g3 is one of the responsible factors for the acceleration of Aβ accumulation through decreased IDE expression. As a robust IDE expression in human astrocytes has been also reported [46], induction of Pla2g3 expression may reduce IDE expression in astrocytes although previous studies of IDE regulation in AD gave inconsistent results, which showed either reduction [47–49] or induction of IDE in AD [50]. We could not exclude the possibility that not only suppression of IDE, but also human Pla2g3-induced inflammation [41] and neuronal cell death [51] would be closely related to the progression of AD pathophysiology. Crossing *APPsw* mice with Pla2g3 knockout mice could be another possible study to comprehend the further role of Pla2g3 involved in AD pathology.

In summary, our data indicate that Pla2g3 plays a pivotal role in suppression of IDE. Thus, increased Pla2g3 expression in the astrocytes by chronic oxidative stress may disrupt Aβ homeostasis, which ultimately leads to the initiation and/or progression of AD. Consequently, the Pla2g3 and IDE pathway could be a suitable target for the development of novel treatment strategies for AD.

## Supporting Information

S1 ARRIVE Checklist Completed “The ARRIVE Guidelines Checklist” for reporting animal data in this manuscript. (PDF)

S1 Fig PLA2s expression in TR-AST and mouse cerebral cortex under oxidative stress. *A*, Quantitative PCR results of PLA2s expression in TR-AST cells treated with 2mM hydrogen peroxide for 6 hours compared with non-treated controls. Fold changes to non-treated controls are indicated. *B*-*D*, Quantitative PCR results of indicated PLA2s in mouse cortex. Fold changes to the aged wild-type mice on normal diet are indicated. n = 4 in each group. Abbreviation used; WT normal; wild type 29 months old mice fed on normal diet, WT def; 29 months old wild type mice fed on Vitamin E deficient diet, ttpKO def; 29 months old *Ttpa* ^*-/-*^ mice fed on Vitamin E deficient diet.
(TIF)

S1 Table Nucleotide sequences of oligonucleotides used in this study. The sequences are shown in 5’ to 3’ direction.
(TIF)

## References

[1] Selkoe DJ. Alzheimer's disease: genes, proteins, and therapy. Physiol Rev. 2001;81(2):741–66. . 11274343 10.1152/physrev.2001.81.2.741

[2] Hardy J, Allsop D. Amyloid deposition as the central event in the aetiology of Alzheimer's disease. Trends Pharmacol Sci. 1991;12(10):383–8. . 1763432 10.1016/0165-6147(91)90609-v

[3] Hardy J, Selkoe DJ. The amyloid hypothesis of Alzheimer's disease: progress and problems on the road to therapeutics. Science. 2002;297(5580):353–6. 10.1126/science.1072994 . 12130773

[4] Katzman R, Saitoh T. Advances in Alzheimer's disease. FASEB J. 1991;5(3):278–86. . 2001787

[5] Brookmeyer R, Evans DA, Hebert L, Langa KM, Heeringa SG, Plassman BL, et al National estimates of the prevalence of Alzheimer's disease in the United States. Alzheimers Dement. 2011;7(1):61–73. 10.1016/j.jalz.2010.11.007 21255744 PMC3052294

[6] Sperling RA, Aisen PS, Beckett LA, Bennett DA, Craft S, Fagan AM, et al Toward defining the preclinical stages of Alzheimer's disease: recommendations from the National Institute on Aging-Alzheimer's Association workgroups on diagnostic guidelines for Alzheimer's disease. Alzheimers Dement. 2011;7(3):280–92. 10.1016/j.jalz.2011.03.003 21514248 PMC3220946

[7] Hsiao K, Chapman P, Nilsen S, Eckman C, Harigaya Y, Younkin S, et al Correlative memory deficits, Abeta elevation, and amyloid plaques in transgenic mice. Science. 1996;274(5284):99–102. . 8810256 10.1126/science.274.5284.99

[8] Kawarabayashi T, Younkin LH, Saido TC, Shoji M, Ashe KH, Younkin SG. Age-dependent changes in brain, CSF, and plasma amyloid (beta) protein in the Tg2576 transgenic mouse model of Alzheimer's disease. J Neurosci. 2001;21(2):372–81. . 11160418 10.1523/JNEUROSCI.21-02-00372.2001 PMC6763819

[9] Barnham KJ, Masters CL, Bush AI. Neurodegenerative diseases and oxidative stress. Nat Rev Drug Discov. 2004;3(3):205–14. 10.1038/nrd1330 . 15031734

[10] Moreira PI, Smith MA, Zhu X, Nunomura A, Castellani RJ, Perry G. Oxidative stress and neurodegeneration. Ann N Y Acad Sci. 2005;1043:545–52. 10.1196/annals.1333.062 . 16037277

[11] Agostinho P, Cunha RA, Oliveira C. Neuroinflammation, oxidative stress and the pathogenesis of Alzheimer's disease. Curr Pharm Des. 2010;16(25):2766–78. . 20698820 10.2174/138161210793176572

[12] Müller WE, Eckert A, Kurz C, Eckert GP, Leuner K. Mitochondrial dysfunction: common final pathway in brain aging and Alzheimer's disease—therapeutic aspects. Mol Neurobiol. 2010;41(2–3):159–71. 10.1007/s12035-010-8141-5 . 20461558

[13] Santos RX, Correia SC, Wang X, Perry G, Smith MA, Moreira PI, et al Alzheimer's disease: diverse aspects of mitochondrial malfunctioning. Int J Clin Exp Pathol. 2010;3(6):570–81. 20661404 PMC2907118

[14] Butterfield DA, Lauderback CM. Lipid peroxidation and protein oxidation in Alzheimer's disease brain: potential causes and consequences involving amyloid beta-peptide-associated free radical oxidative stress. Free Radic Biol Med. 2002;32(11):1050–60. . 12031889 10.1016/s0891-5849(02)00794-3

[15] Yokota T, Igarashi K, Uchihara T, Jishage K, Tomita H, Inaba A, et al Delayed-onset ataxia in mice lacking alpha -tocopherol transfer protein: model for neuronal degeneration caused by chronic oxidative stress. Proc Natl Acad Sci U S A. 2001;98(26):15185–90. 10.1073/pnas.261456098 11752462 PMC65004

[16] Nishida Y, Ito S, Ohtsuki S, Yamamoto N, Takahashi T, Iwata N, et al Depletion of vitamin E increases amyloid beta accumulation by decreasing its clearances from brain and blood in a mouse model of Alzheimer disease. J Biol Chem. 2009;284(48):33400–8. 10.1074/jbc.M109.054056 19679659 PMC2785184

[17] Nishida Y, Yokota T, Takahashi T, Uchihara T, Jishage K, Mizusawa H. Deletion of vitamin E enhances phenotype of Alzheimer disease model mouse. Biochem Biophys Res Commun. 2006;350(3):530–6. 10.1016/j.bbrc.2006.09.083 . 17026966

[18] Lovell MA, Ehmann WD, Butler SM, Markesbery WR. Elevated thiobarbituric acid-reactive substances and antioxidant enzyme activity in the brain in Alzheimer's disease. Neurology. 1995;45(8):1594–601. . 7644059 10.1212/wnl.45.8.1594

[19] Feng L, Long HY, Liu RK, Sun DN, Liu C, Long LL, et al A quantum dot probe conjugated with aβ antibody for molecular imaging of Alzheimer's disease in a mouse model. Cell Mol Neurobiol. 2013;33(6):759–65. 10.1007/s10571-013-9943-6 . 23695800 PMC11497885

[20] Ichijo M, Ishibashi S, Li F, Yui D, Miki K, Mizusawa H, et al Sphingosine-1-Phosphate Receptor-1 Selective Agonist Enhances Collateral Growth and Protects against Subsequent Stroke. PLoS One. 2015;10(9):e0138029 10.1371/journal.pone.0138029 26367258 PMC4569572

[21] Ajioka I, Jinnou H, Okada K, Sawada M, Saitoh S, Sawamoto K. Enhancement of neuroblast migration into the injured cerebral cortex using laminin-containing porous sponge. Tissue Eng Part A. 2015;21(1–2):193–201. 10.1089/ten.TEA.2014.0080 . 25010638

[22] Yui D, Yoneda T, Oono K, Katayama T, Imaizumi K, Tohyama M. Interchangeable binding of Bcl10 to TRAF2 and cIAPs regulates apoptosis signaling. Oncogene. 2001;20(32):4317–23. . 11466612 10.1038/sj.onc.1204576

[23] Tetsuka K, Hosoya KI, Ohtsuki S, Takanaga H, Yanai N, Ueda M, et al Acidic amino acid transport characteristics of a newly developed conditionally immortalized rat type 2 astrocyte cell line (TR-AST). Cell Struct Funct. 2001;26(4):197–203. . 11699636 10.1247/csf.26.197

[24] Butenko O, Dzamba D, Benesova J, Honsa P, Benfenati V, Rusnakova V, et al The increased activity of TRPV4 channel in the astrocytes of the adult rat hippocampus after cerebral hypoxia/ischemia. PLoS One. 2012;7(6):e39959 10.1371/journal.pone.0039959 22761937 PMC3384594

[25] Sanchez-Ortiz E, Yui D, Song D, Li Y, Rubenstein JL, Reichardt LF, et al TrkA gene ablation in basal forebrain results in dysfunction of the cholinergic circuitry. J Neurosci. 2012;32(12):4065–79. 10.1523/JNEUROSCI.6314-11.2012 22442072 PMC3403817

[26] Li Y, Yui D, Luikart BW, McKay RM, Rubenstein JL, Parada LF. Conditional ablation of brain-derived neurotrophic factor-TrkB signaling impairs striatal neuron development. Proc Natl Acad Sci U S A. 2012;109(38):15491–6. 10.1073/pnas.1212899109 22949667 PMC3458400

[27] Sato H, Taketomi Y, Isogai Y, Miki Y, Yamamoto K, Masuda S, et al Group III secreted phospholipase A2 regulates epididymal sperm maturation and fertility in mice. J Clin Invest. 2010;120(5):1400–14. 10.1172/JCI40493 20424323 PMC2860917

[28] Dennis EA. Diversity of group types, regulation, and function of phospholipase A2. J Biol Chem. 1994;269(18):13057–60. . 8175726

[29] Dennis EA. The growing phospholipase A2 superfamily of signal transduction enzymes. Trends Biochem Sci. 1997;22(1):1–2. . 9020581 10.1016/s0968-0004(96)20031-3

[30] Smalheiser NR, Dissanayake S, Kapil A. Rapid regulation of neurite outgrowth and retraction by phospholipase A2-derived arachidonic acid and its metabolites. Brain Res. 1996;721(1–2):39–48. . 8793082 10.1016/0006-8993(96)00134-5

[31] Ikeno Y, Konno N, Cheon SH, Bolchi A, Ottonello S, Kitamoto K, et al Secretory phospholipases A2 induce neurite outgrowth in PC12 cells through lysophosphatidylcholine generation and activation of G2A receptor. J Biol Chem. 2005;280(30):28044–52. 10.1074/jbc.M503343200 . 15927955

[32] Masuda S, Murakami M, Takanezawa Y, Aoki J, Arai H, Ishikawa Y, et al Neuronal expression and neuritogenic action of group X secreted phospholipase A2. J Biol Chem. 2005;280(24):23203–14. 10.1074/jbc.M500985200 . 15781456

[33] Schaeffer EL, da Silva ER, Novaes BeA, Skaf HD, Gattaz WF. Differential roles of phospholipases A2 in neuronal death and neurogenesis: implications for Alzheimer disease. Prog Neuropsychopharmacol Biol Psychiatry. 2010;34(8):1381–9. 10.1016/j.pnpbp.2010.08.019 . 20804810

[34] Yagami T, Ueda K, Asakura K, Hata S, Kuroda T, Sakaeda T, et al Human group IIA secretory phospholipase A2 induces neuronal cell death via apoptosis. Mol Pharmacol. 2002;61(1):114–26. . 11752212 10.1124/mol.61.1.114

[35] Yagami T, Ueda K, Asakura K, Nakazato H, Hata S, Kuroda T, et al Human group IIA secretory phospholipase A2 potentiates Ca2+ influx through L-type voltage-sensitive Ca2+ channels in cultured rat cortical neurons. J Neurochem. 2003;85(3):749–58. . 12694401 10.1046/j.1471-4159.2003.01712.x

[36] Kriem B, Sponne I, Fifre A, Malaplate-Armand C, Lozac'h-Pillot K, Koziel V, et al Cytosolic phospholipase A2 mediates neuronal apoptosis induced by soluble oligomers of the amyloid-beta peptide. FASEB J. 2005;19(1):85–7. 10.1096/fj.04-1807fje . 15486059

[37] Sanchez-Mejia RO, Newman JW, Toh S, Yu GQ, Zhou Y, Halabisky B, et al Phospholipase A2 reduction ameliorates cognitive deficits in a mouse model of Alzheimer's disease. Nat Neurosci. 2008;11(11):1311–8. 10.1038/nn.2213 18931664 PMC2597064

[38] Chiricozzi E, Fernandez-Fernandez S, Nardicchi V, Almeida A, Bolaños JP, Goracci G. Group IIA secretory phospholipase A2 (GIIA) mediates apoptotic death during NMDA receptor activation in rat primary cortical neurons. J Neurochem. 2010;112(6):1574–83. 10.1111/j.1471-4159.2010.06567.x . 20067579

[39] Venkateshappa C, Harish G, Mahadevan A, Srinivas Bharath MM, Shankar SK. Elevated oxidative stress and decreased antioxidant function in the human hippocampus and frontal cortex with increasing age: implications for neurodegeneration in Alzheimer's disease. Neurochem Res. 2012;37(8):1601–14. 10.1007/s11064-012-0755-8 22461064

[40] Sato H, Kato R, Isogai Y, Saka G, Ohtsuki M, Taketomi Y, et al Analyses of group III secreted phospholipase A2 transgenic mice reveal potential participation of this enzyme in plasma lipoprotein modification, macrophage foam cell formation, and atherosclerosis. J Biol Chem. 2008;283(48):33483–97. 10.1074/jbc.M804628200 18801741 PMC2662271

[41] Sato H, Taketomi Y, Isogai Y, Masuda S, Kobayashi T, Yamamoto K, et al Group III secreted phospholipase A2 transgenic mice spontaneously develop inflammation. Biochem J. 2009;421(1):17–27. 10.1042/BJ20082429 19371233 PMC2708930

[42] Taketomi Y, Ueno N, Kojima T, Sato H, Murase R, Yamamoto K, et al Mast cell maturation is driven via a group III phospholipase A2-prostaglandin D2-DP1 receptor paracrine axis. Nat Immunol. 2013;14(6):554–63. 10.1038/ni.2586 23624557 PMC4065307

[43] DeCoster MA. Group III secreted phospholipase A2 causes apoptosis in rat primary cortical neuronal cultures. Brain Res. 2003;988(1–2):20–8. . 14519523 10.1016/s0006-8993(03)03326-2

[44] Edbauer D, Willem M, Lammich S, Steiner H, Haass C. Insulin-degrading enzyme rapidly removes the beta-amyloid precursor protein intracellular domain (AICD). J Biol Chem. 2002;277(16):13389–93. 10.1074/jbc.M111571200 . 11809755

[45] Murakami M, Taketomi Y. Secreted phospholipase A2 and mast cells. Allergol Int. 2015;64(1):4–10. 10.1016/j.alit.2014.07.005 . 25572553

[46] Mulder SD, Veerhuis R, Blankenstein MA, Nielsen HM. The effect of amyloid associated proteins on the expression of genes involved in amyloid-β clearance by adult human astrocytes. Exp Neurol. 2012;233(1):373–9. 10.1016/j.expneurol.2011.11.001 . 22101005

[47] Pérez A, Morelli L, Cresto JC, Castaño EM. Degradation of soluble amyloid beta-peptides 1–40, 1–42, and the Dutch variant 1-40Q by insulin degrading enzyme from Alzheimer disease and control brains. Neurochem Res. 2000;25(2):247–55.. 10786709 10.1023/a:1007527721160

[48] Cook DG, Leverenz JB, McMillan PJ, Kulstad JJ, Ericksen S, Roth RA, et al Reduced hippocampal insulin-degrading enzyme in late-onset Alzheimer's disease is associated with the apolipoprotein E-epsilon4 allele. Am J Pathol. 2003;162(1):313–9. 12507914 10.1016/s0002-9440(10)63822-9 PMC1851126

[49] Zhao Z, Xiang Z, Haroutunian V, Buxbaum JD, Stetka B, Pasinetti GM. Insulin degrading enzyme activity selectively decreases in the hippocampal formation of cases at high risk to develop Alzheimer's disease. Neurobiol Aging. 2007;28(6):824–30. 10.1016/j.neurobiolaging.2006.05.001 . 16769157

[50] Dorfman VB, Pasquini L, Riudavets M, López-Costa JJ, Villegas A, Troncoso JC, et al Differential cerebral deposition of IDE and NEP in sporadic and familial Alzheimer's disease. Neurobiol Aging. 2010;31(10):1743–57. 10.1016/j.neurobiolaging.2008.09.016 19019493 PMC3266723

[51] Martínez-García A, Sastre I, Recuero M, Aldudo J, Vilella E, Mateo I, et al PLA2G3, a gene involved in oxidative stress induced death, is associated with Alzheimer's disease. J Alzheimers Dis. 2010;22(4):1181–7. 10.3233/JAD-2010-101348 . 20930276

