# Supplementary figures and images for: Enhanced Phospholipase A2 Group 3 Expression by Oxidative Stress Decreases the Insulin-Degrading Enzyme
Source: PLoS One. 2015 Dec 4;10(12):e0143518. doi: 10.1371/journal.pone.0143518 (PMC4670075; doi:10.1371/journal.pone.0143518)

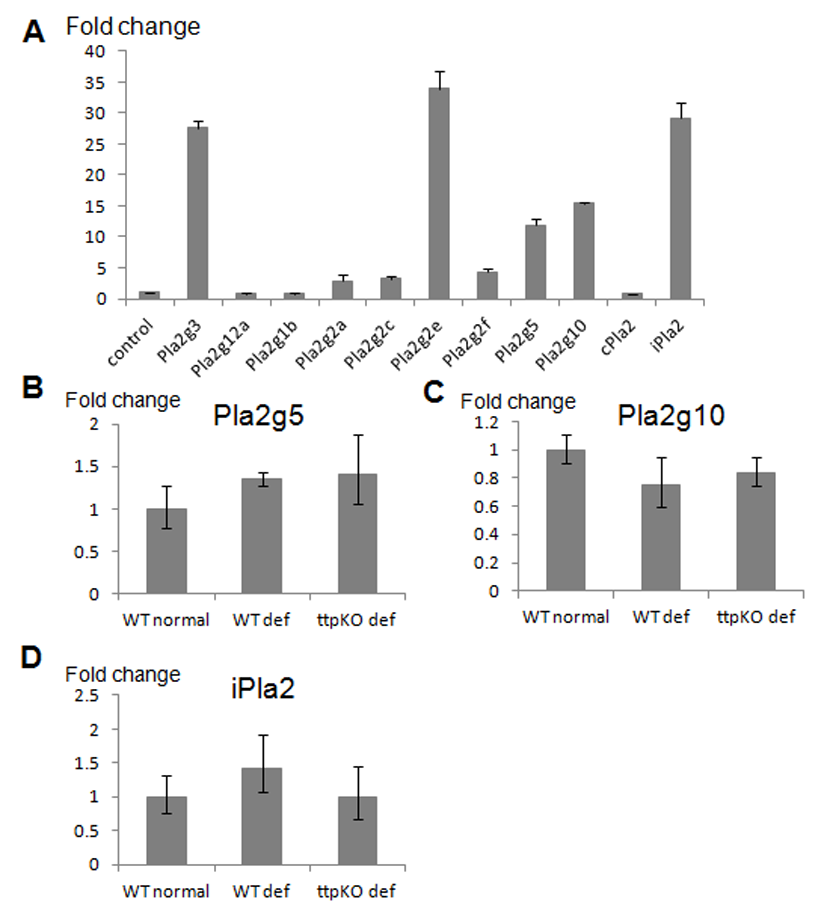

Supplement: S1 Fig — A, Quantitative PCR results of PLA2s expression in TR-AST cells treated with 2mM hydrogen peroxide for 6 hours compared with non-treated controls. Fold changes to non-treated controls are indicated. B-D, Quantitative PCR results of indicated PLA2s in mouse cortex. Fold changes to the aged wild-type mice on normal diet are indicated. n = 4 in each group. Abbreviation used; WT normal; wild type 29 months old mice fed on normal diet, WT def; 29 months old wild type mice fed on Vitamin E deficient diet, ttpKO def; 29 months old Ttpa -/- mice fed on Vitamin E deficient diet. (TIF) [file pone.0143518.s002.tif]

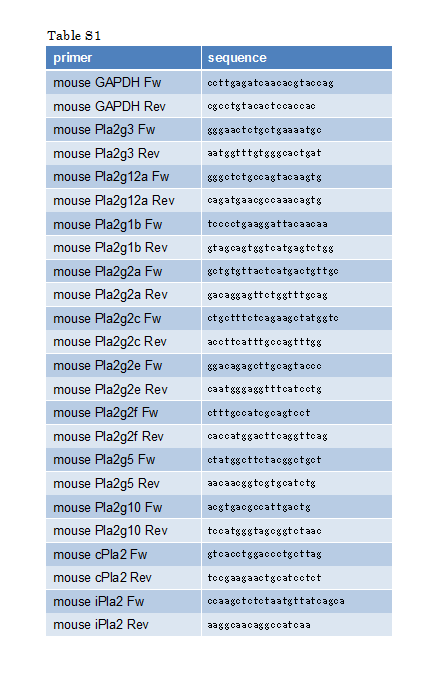

Supplement: S1 Table — The sequences are shown in 5’ to 3’ direction. (TIF) [file pone.0143518.s003.tif]
